# For optimal treatment of musculoskeletal pain among elderly individuals, clarification of its aetiology is essential

**DOI:** 10.51866/lte.499

**Published:** 2024-05-21

**Authors:** Josef Finsterer

**Affiliations:** 1 MD, PhD, Neurology and Neurophysiology Center, Postfach 20, Vienna, Austria., Email: fifigs1@yahoo.de

**Keywords:** Myalgia, Arthralgia, Musculoskeletal pain, Analgesics, Depression

**Dear editor,**

I read with interest the article by Sengupta et al. on a mixed-method study of 255 elderly patients with musculoskeletal pain (MSP) evaluated using a structured questionnaire.^1^ The study showed that 57% of the enrolled patients reported MSP, with the knee joint being the most commonly affected site. Multivariate analysis revealed that comorbidity, age, depression and drug use were associated with MSP It was concluded that the treatment of MSP should focus on managing comorbidities, providing mental support and informing about the side effects of over-the-counter analgesics. The study is valuable but has concerning limitations that should be discussed.

The main limitation of the study is that only symptoms were assessed; their cause was not explored. It is imperative to determine the underlying aetiology to adequately treat MSP For example, MSP due to osteoporosis may favourably respond to osteoporosis treatment, statin-induced myalgia to statin withdrawal and rheumatoid arthritis to anti-inflammatory drugs. While I agree with the conclusions, elucidating the underlying cause of MSP is more important than treating comorbidities, providing mental support and educating about the side effects of over-the-counter analgesics. Only when the aetiology is clarified can optimal therapy be carried out.

Another limitation of the study is that MSP was not defined.^1^ It is crucial to determine whether the authors meant pain due to an orthopaedic, a neurological or a rheumatological problem. The definition of chronic MSP was also unclear. Accordingly, the inclusion and exclusion criteria must be defined to ensure a homogeneous study population.

The study also did not report the current medications taken in addition to analgesics. Since various drugs can trigger MSP,^2^ it is important to know what drugs the patients were currently taking in addition to the medications for MSP Some drugs known to cause MSP include steroids, heparin, metformin, zolpidem, clonazepam, alprazolam, tramadol, omeprazol, statins, levofloxacin, bisphosphonates, anastrozole, exemestane, letrozole and isoretinoin.

The relevance of the study to practice and the clinical implications of the results were not described. Given that 57% of the enrolled patients experienced MSP, there is an urgent need to improve the diagnostic and therapeutic management for these patients.

In summary, the study offers valuable insights but has limitations that raise concerns regarding the results and their interpretation. Addressing these issues could strengthen the conclusions and improve the status of the study. A healthcare system that enables adequate diagnosis of MSP and provides the best possible therapy for the underlying disease must be created to optimise the care of elderly patients with MSP If depression is a cause of patient complaints, it must be treated adequately with medication.
